# Pancreatic Cancer in Celiac Disease Patients—A Systematic Review and Meta-Analysis

**DOI:** 10.3390/ijerph20021565

**Published:** 2023-01-14

**Authors:** Iga Gromny, Katarzyna Neubauer

**Affiliations:** Division of Dietetics, Department of Gastroenterology and Hepatology, Wroclaw Medical University, Borowska 213, 50-556 Wroclaw, Poland

**Keywords:** pancreatic cancer, celiac disease, autoimmune diseases

## Abstract

Background: Celiac disease (CD) is an autoimmune enteropathy affecting approximately 1% of the population and is associated with an increased risk of enteropathy-associated T-cell lymphoma and small bowel adenocarcinoma, whereas the association between CD and other malignancies is unclear. Since pancreatic cancer (PC) remains one of the most lethal neoplasms and its incidence is increasing despite numerous ongoing research on diagnostic biomarkers and novel therapies, we aimed to investigate whether CD has an impact on the risk of PC. Material and Methods: We performed a systematic review of the literature published from January 2000 to March 2022 in two databases: Web of Science and Scopus and a meta-analysis of eligible studies. Results: Our search identified eight publications included in the systematic review. A total of five studies involving 47,941 patients, including 6399 CD patients with malignancies and 1231 PC cases were included in the meta-analysis and 221 cases of PC in CD patients with other cancers were recognized. The pooled OR for PC was 1.46 (95% CI 1.26–1.7) with significant heterogeneity (89.1%; *p* < 0.05), suggesting that CD patients with malignancies were at higher risk for PC. Conclusions: The association between CD and PC is uncertain. However, the results of the current meta-analysis may indicate an increased risk of PC in the group of patients with CD and other cancers. Further multicenter studies are warranted.

## 1. Introduction

Celiac disease (CD) is an immune-mediated disorder that primarily affects the small intestine and occurs in genetically predisposed individuals with the presence of leukocyte antigen HLA-DQ2/HLA-DQ8 haplotypes [1]. Consumption of gluten in susceptible individuals contributes to inflammation of the small intestinal mucosa and villous atrophy [2]. The worldwide seroprevalence and prevalence of CD were recently estimated to be 1.4% and 0.7%, respectively [3]. However, approximately 70% of cases remain undiagnosed and untreated [4]. CD can occur at any age, but the highest prevalence rates have been observed in children [5]. The incidence of CD has increased by 7.5% per year in recent decades and CD has become a significant health problem worldwide [3]. The increase in new diagnoses is due to growing awareness of the disease and better availability of testing, but also because the increase in actual incidence is a result of environmental changes that may promote loss of tolerance to gluten in the diet [4,6,7]. Diagnostic criteria for CD have evolved, yet there is a high degree of agreement among guidelines published over the past few decades [8]. Despite the intensive development of new treatment strategies [9], a gluten-free diet (GFD) is the only available, but not universally effective, therapeutic option for CD patients. CD shows a wide spectrum of intestinal and extraintestinal manifestations and may be associated with other autoimmune diseases such as type 1 diabetes, inflammatory bowel diseases and genetic disorders. The relationship between CD and malignancies is still under investigation. Enteropathy-associated T-cell lymphoma (relative risk, (RR) = 3.1) and small bowel adenocarcinoma (RR = 3.05) have been for decades the best-known malignancies connected to CD [10]. However, the relationship between CD and other types of neoplasms is still unclear [11,12].

At the same time, cancer is one of the leading causes of death worldwide and will be responsible for nearly 10 million deaths in 2020 [13], a position shared with malnutrition and hunger which kill almost 10 million people each year, amongst whom three million are children [14]. Pancreatic cancer (PC) is the seventh leading cause of cancer-related deaths worldwide due to its extremely poor prognosis [15]. Based on GLOBOCAN data 458,918 new cases of PC were reported worldwide in 2018, accounting for 2.5% of all cancers. Moreover, PC is becoming an increasingly common cause of cancer mortality and a 2.3-fold increase in the global number of cases and deaths from PC has been demonstrated [16]. The incidence of PC varies by region and population group. The highest age-standardized rate incidence was observed in Europe (7.7 per 100,000 population) and North America (7.6 per 100,000), while the lowest was observed in Africa (2.2 per 100,000 population). Varying exposure to risk factors may explain these differences between regions [17,18]. However, the lowest prevalence in Africa may be due to the effects of malnutrition and starvation on that continent, where people die of hunger before exposure resulting in PC [19,20]. PC is more common in men than in women. Noticeably, the incidence rate increases with age in both sexes. The highest incidence occurs in women aged 75–79 years and in men aged 65–69 years [17]. Known risk factors for PC include tobacco smoking, diabetes mellitus, obesity, dietary factors, alcohol abuse, age, ethnicity, family history and genetic factors, Helicobacter pylori infection, non-O blood group and chronic pancreatitis, while inherited risk factors contribute to 5–10% of PC cases [17]. Dietary factors are estimated to have an impact on up to 30–50% of PC cases [21,22]. Early diagnosis and treatment of PC remain a formidable challenge. Approximately one-third of patients have locally advanced disease and 50% of patients are found to have metastatic disease at the time of diagnosis [23].

Our systematic review and meta-analysis investigated the possible relationship between CD and PC. There were three phenomena motivating us to conduct this analysis: 1. The explosion of new cases of CD and PC. 2. The known association of CD with intestinal tumors and suggested connection of CD to other tumors. 3. CD–related factors, mainly immunity-related [24] and metabolic [25,26,27], that may potentially impact PC development. Therefore, we attempted to conduct a systematic review of the current knowledge on the association between CD and PC and a meta-analysis of the available studies. Clarification and better understanding of such an association could have clinical implications and guide the future direction of research on CD and PC.

## 2. Materials and Methods

### 2.1. Protocol and Search Strategy

A systematic review was conducted in March 2022 and included manuscripts published from January 2000 to March 2022 in English. Two respective databases, Web of Science and Scopus were queried. To prepare queries, the following expressions ‘‘celiac disease’’ OR ‘‘coeliac disease’’ AND ‘‘cancer*’’ OR ‘‘tumor*’’ OR ‘‘carcinoma*’’ OR ‘‘neoplasm*’’ AND “pancreas” OR “pancreatic” were used. The asterisks allowed us to retrieve records where query words appeared with suffixes (e.g., cancer|s). The reporting of this systematic review was guided by the standards of the Preferred Reporting Items for Systematic Review and Meta-Analysis (PRISMA) Statement [28]. The review was not registered.

### 2.2. Eligibility Criteria

Cohort studies that reported PC in patients with CD were reviewed and included if eligible. The following studies were excluded: experimental studies, animal studies, editorials, conference papers and book chapters.

### 2.3. Study Selection and Data Extraction

Study selections were performed by the authors based on the eligibility criteria. Any disagreement on study selection between two authors was discussed for consensus. Firstly, the potentially relevant studies were identified from two databases; then, duplicates were removed. Secondly, the remaining studies’ titles and abstracts were screened; unrelated studies were excluded. Thirdly, the remaining studies were examined for full texts; unrelated studies were excluded. Finally, eligible studies were included for further review. Data from the included studies were independently extracted into the pilot standardized datasheet by two authors. The following data were extracted from each study: author, year of publication, study site, study design, number of patients with CD, number of patients with PC, main findings, characteristics of the participants, age and gender.

### 2.4. Quality of the Included Studies

Quality assessment of the selected studies was performed using the Newcastle–Ottawa Quality Assessment Scale (NOS) [29]. NOS is scored by awarding points for fulfilling criteria related to selection (representativeness of the exposed cohort, selection of the non-exposed cohort, ascertainment of exposure, a demonstration that outcome of interest was not present at the start of study, comparability (comparability of cohorts based on the design or analysis) and outcomes of studies. Possible totals are 4 points for selection, 2 points for comparability and 3 points for outcomes.) Both authors (I.G. and K.N.) independently performed data abstraction and quality assessment. Discrepancies between authors’ assessments were discussed until a consensus was reached.

### 2.5. Statistical Analysis

EPIINFO version 7.2.4.0 (The Centers for Disease Control and Prevention, Atlanta, GA, USA) and Meta Win version 3.0.6 (Center for Biological Data Science, Richmond, VA, USA) beta were used for the statistical analysis. The overall odds ratio (OR) and relative risk (RR) were pooled using a random-effects model. Ors and RRs of each study, at 95% confidence intervals (CIs), were calculated and Mantel–Haenszel test was conducted. I^2^ statistics with CIs were used to assess statistical heterogeneity. Statistical significance was set at *p* < 0.05.

## 3. Results

### 3.1. Search Results

**Figure 1:**
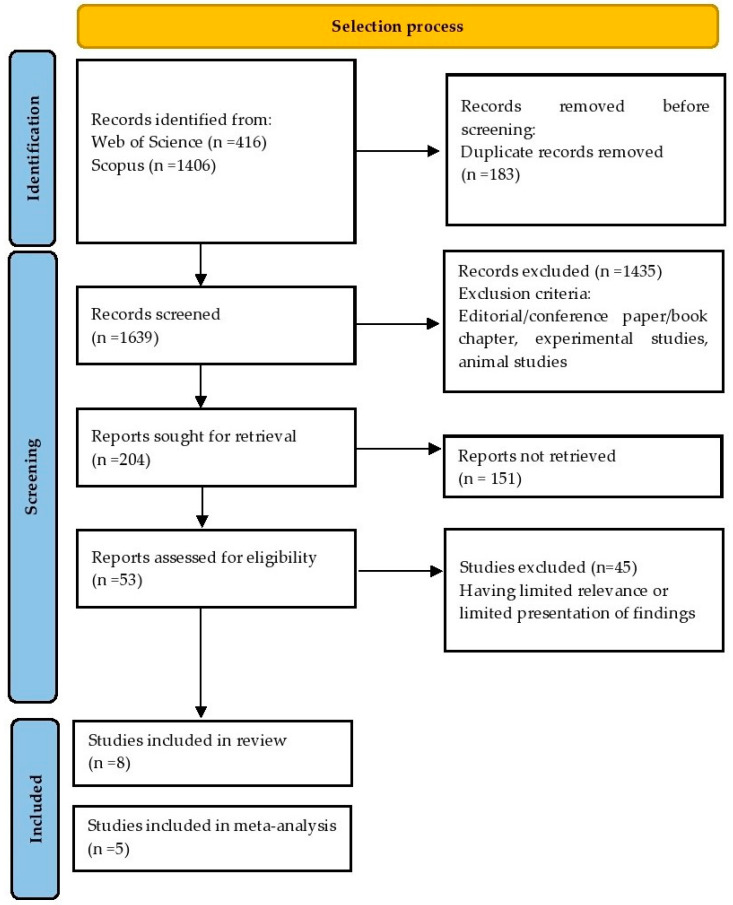
Flowchart presenting the selection process.

Our primary search using the selected key phrases resulted in 1822 publications, of which 1639 entered the title screening phase. Exclusion criteria removed 1435 entries leaving 204 records in our database for full-text reading. Cross-search and a manual search revealed an additional two eligible articles. Finally, this systematic review was prepared based on eight publications. Five studies were included in the meta-analysis. The selection process is presented in Figure 1.

### 3.2. Quality Assessment

All the studies proved appropriate representativeness of the exposed cohort and appropriate selection of the non-exposed cohort. No study could prove ascertainment of exposure. Two studies [30,31] showed no outcome of interest at the start of the study. All studies were controlled for confounders. All studies demonstrated assessment for the outcome and follow-up that was long enough for outcomes. One study [30] showed adequacy of follow-up cohort (Table 1).

### 3.3. Celiac Disease and Pancreatic Cancer

In recent decades, several studies have investigated cancer risk in CD patients. Only a few studies have examined this risk in relation to PC. Our search revealed eight papers that included data on PC in CD. The characteristics of the studies included in the systematic review are presented in Table 2.

Several papers on malignancies in CD did not report data on PC [38,39,40,41,42,43]. In the study in the Lothian region of Scotland, the overall risk of malignancy in CD decreased with time after diagnosis and was not significantly increased after 15 years. The study included data on esophagal and colorectal tumors as well as general GI cancers, yet PC was not specifically addressed [38]. Further, in the study of Green et al. on 381 CD patients (43 cases of cancer), PC was not recognized [39]. In another study on the risk of malignancy and mortality in CD among 4732 people with CD compared with 23,620 matched controls there were 134 patients with at least one malignancy; however, the GI tumors were combined in one group. The authors concluded that people with CD have a modest increase in overall risks of malignancy and mortality. Similar to other studies, most of this excess risk occurred in the year of follow-up after diagnosis of CD [41]. In the study of Viljamaa, the prevalence of malignancies was similar to that in the general population [42]. An Italian study suggested an increased risk of cancer related to age [44].

### 3.4. Meta-Analysis

**Figure 2:**
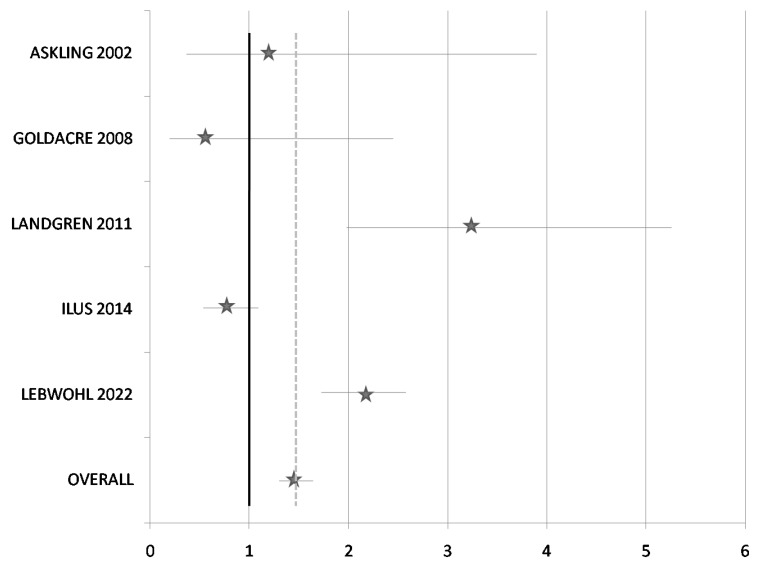
Forest plot for the association between celiac disease and pancreatic cancer [30,32,34,35,36].

A total of five studies were included in the meta-analysis (Table 3). All five studies with 47,941 patients, including 6399 patients with CD and malignancies and 1213 PC cases, including 221 cases in CD patients and other cancers, were recognized. The pooled OR for PC was 1.46 (95% CI 1.26–1.7) and the pooled RR was 1.4 (95% CI 1.25–1.67), suggesting that patients with CD had a slightly higher risk of PC. Figure 2 shows the forest plot of the association between CD and PC. However, a high degree of heterogeneity was found (I^2^ = 89.1%; *p* < 0.05). Subgroup analysis was not possible due to the small number of studies included in our meta-analysis. In consequence, the results should be prudently considered and more detailed studies with adequate sample size are needed to determine the association between CD and the risk of PC.

## 4. Discussion

CD is associated with an increased risk of mortality [45] and several types of malignancies [30]. The results of available studies on the risk of PC in CD are inconsistent and vary from the studies demonstrating the increased risk of PC in CD patients [30,31,32,35] to research showing that PC risk and mortality were not increased [33,34,37] and one study in which the risk of PC was decreased [35]. The results of our meta-analysis of five eligible studies suggest, that the risk of PC in CD patients with other cancers might be increased. However, this finding should be interpreted with caution, primarily because of the lack of homogeneity in the pooled data.

All of the available studies, with one exception, have been performed in Europe. All European studies have been carried out in the northern part of the continent and most of the studies (five out eight) are in Scandinavian countries (Finland, Sweden). In turn, the only study carried out outside Europe involved a cohort restricted to males [35]. This might be seen as its limitation, as PC is more common in men [17], whereas CD is more common in women [3]. Considering the geographic diversity in the prevalence of both CD [5] and PC [17], the results of studies conducted in one world region cannot be directly extrapolated to a global extension.

The length of the observation influenced the risk of malignancies in CD patients. However, here also the results of the studies could have been more consistent. For instance, the study by Elfstrom et al. [31] demonstrated the increased risk only in the first year after diagnosis. In the study by Askling et al. [32], risk was reduced with time. Authors found out that adult patients with CD had an increased overall risk of cancer (SIR = 1.3; 95% CI, 1.2–1.5); however, the RR declined with duration of follow-up and was only slightly or even insignificantly increased after 10 years, reaching SIR of 1.1 [33]. Furthermore, Lebwohl et al. observed the association between CD and cancer only in the first year after diagnosis (HR, 2.47; 95% CI, 2.22–2.74). This might be partially explained by the adherence to GFD and its protective effect on cancer in patients with CD. On the contrary, it may be due to the increased surveillance and medical care among CD patients. The strongest association between CD and cancer was noted in hematologic cancers (HR, 1.90; 95% CI, 1.70–2.13), lymphoproliferative cancers (HR, 2.20; 95% CI, 1.94–2.49) and GI cancers (HR, 1.34; 95%CI, 1.24–1.45). Among the last subtype of cancer, the risk of PC (HR, 2.30; 95% CI, 1.87–2.82) and hepatobiliary cancer (HR, 1.80; 95% CI, 1.44–2.25) were increased. Interestingly, the elevated risk of PC (HR, 1.66; 95% CI, 1.32–2.10) and hepatobiliary cancer (HR, 1.61; 95% CI, 1.26–2.05) persisted in long-term observation [30]. To avoid bias resulting from inclusion of the cases being a cause for the work-up leading to the diagnosis of CD, or having been recognized during the work-up for CD, authors excluded the first year after CD diagnosis [32] or calculated the risk including and excluding the first year after diagnosis [34]. In turn, in a population-based, nationwide case-control study by van Gils et al. [46], authors took a different approach and assessed the risk of lymphomas and GI adenocarcinomas in newly diagnosed CD patients. They did not report data on PC, but found out, that enteropathy-associated T-cell lymphoma, lymphoma and CD were often synchronously diagnosed. In turn, in the study by Ilus et al. patients were stratified according to the duration of follow-up (<2 years (21,508), 2–4.9 years (21,556) and ≥5 years (7794). 1626 cancers occurred among CD patients, while 1735 were expected (SIR 0.94; 95% CI 0.89–0.98). Compared with the general population, the overall risk of malignancy was not increased, yet SIR was increased after 5 years from CD diagnosis. 45 cases of PC were observed, whereas 62 were expected, resulting in decreased SIR for PC. The risk was decreased, especially in females (Table 2). [36]. This may reflect smaller exposure among females to the other risk factors for PC, such as obesity or smoking; however, it was not directly assessed by the study. In turn, Lebwohl et al. observed, that cancer risk in CD was elevated in men but not women [30]. The other significant risk factor for cancers is age. This is crucial as there is a rise in CD cases in older groups of patients [47]. It was shown that the increased risk of cancer in CD patients was mostly limited to patients above the age of 40 [30]. Furthermore, the highest risk for T-cell lymphoma was observed in males between the ages of 50 and 80 when CD was diagnosed at age 50 [46]. Authors noticed, that risk increase was confined to patients diagnosed with CD after age 40 and is primarily present within the first year of diagnosis [37], which corresponds with the findings of Lebwohl et al. [30]. In this study, the overall risk was highest in individuals diagnosed with CD after the age of 60 years (HR, 1.22; 95% CI, 1.16–1.29). Such an observation regarding the risk of PC was not demonstrated in the available studies.

Furthermore, as CD often remains undiagnosed, studies have practically no chance of establishing a cohort covering all patients with CD. Most often, studies identify CD patients from the registries of hospital discharges. Therefore, they may include CD patients with more severe diseases or complications. On the other hand, the prevalence of CD among patients with cancer is unknown and, similar to in the general population, it can be underestimated [48]. It remains challenging to determine if cancer was developing in unrecognized CD or CD developed after cancer diagnosis or cancer therapy exposure.

Summarizing, the available data on the risk of PC in patients with CD are inconsistent and do not allow the derivation of a true correlation between CD and PC.

### Limitations

Our study has several limitations. We searched two databases, Web of Science and Scopus and, although there is literature supporting the choice of these databases [49], this may be considered as a potential limitation. Furthermore, the search was limited to papers published since 1 January 2000 and, while this in our opinion was justified by the available literature demonstrating that there are no earlier papers on the risk of PC in CD patients, it may be interpreted as a limitation. Furthermore, the meta-analysis revealed a lack of homogeneity in the pooled data, which reduces confidence in the results. Therefore, the results should be interpreted with caution. Additionally, there are also limitations related to the analyzed studies demonstrated in the quality assessment. No study could prove the ascertainment of exposure. Two out of five studies showed no outcome of interest at the start of the study. Only one study showed the adequacy of a follow-up cohort. In one of the studies [32] reporting increased risk for PC, a wide CI for SIR of PC leaves considerable doubt about the true SIR and may indicate an unstable statistic. Furthermore, the majority were performed in northern Europe and, considering the geographic variation in the occurrence of both PC and CD, their results cannot be extrapolated to the global population. Studies incorporated into our review differ in methodology and studied populations (prospective and retrospective design, general population-based, hospital-based, different duration of observation). The other issue which has to be considered is the small number of PC cases in CD patients reported in three studies. There were two cases of PC in two studies [33,34] and nine cases in one study [32]. A small sample of participants with PC may cause difficulties in the interpretation of results. With three exceptions [30,32,35], comorbidities were not reported. Concomitant diseases such as diabetes, pancreatitis, obesity and non-alcoholic fatty liver disease may impact PC risk. Finally, a significant number of CD cases remain undiagnosed. Therefore the studies have no prospect of covering all CD patients as the number of patients with an established diagnosis is lower than the serological prevalence of the disease. Moreover, patients, while being diagnosed with cancer or with a diagnosis of cancer, may undergo biopsies that would reveal CD, thus inducing a bias.

## 5. Conclusions

The available data on the risk of PC in patients with CD remain inconsistent and do not allow the deduction of a real correlation between CD and PC. The meta-analysis performed may suggest an increased risk of PC in CD patients with malignancies. However, cautious interpretation of the results is warranted because of the lack of homogeneity of the pooled data. Yet a recent large cohort study suggested the increased risk of PC in CD patients. Further prospective studies, for instance, designed as international projects, are requested to clarify and better understand the complex and still unclear relationship between PC and CD.

## Figures and Tables

**Table 1:** Quality assessment of the selected studies using the Newcastle–Ottawa Quality Assessment Scale for cohort studies.

Study		Askling et al. 2002 [32]	Card et al. 2004 [33]	Goldacre et al. 2008 [34]	Landgren et al. 2011 [35]	Elfstrom et al. 2012 [31]	Ilus et al. 2014 [36]	Koskinen et al. 2020 [37]	Lebwohl et al. 2022 [30]
SELECTION (maximum four)	Representativeness of The Exposed Cohort	1	1	1	1	1	1	1	1
	Selection of The Non-Exposed Cohort	1	1	1	1	1	1	1	1
	Ascertainment of Exposure	0	0	0	0	0	0	0	0
	No Outcome of Interest at Start of The Study	0	0	0	0	1	0	0	1
COMPARABILITY (maximum two)	Controlled for confounders	2	2	2	2	2	2	2	2
OUTCOME (maximum three)	Assessment of outcome	1	1	1	1	1	1	1	1
	Follow-Up Long Enough for Outcomes	1	1	1	1	1	1	1	1
	Adequacy of Follow Up of Cohorts	0	0	0	0	0	0	0	1
Score		6	6	6	6	7	6	6	8

**Table 2:** Characteristics of the studies included in the systematic review and/or meta-analysis.

Author Year Country	Study Design	CD/PC (*n*)	Age and Gender of Participants	Inclusion Criteria	Exclusion Criteria	Relevant Comorbidities	Main Findings
Askling et al. 2002 [32] Sweden	A population-based prospective cohort study	11,019/9	All age categories (0–60+) 6521 F 4498 M	Individuals discharged at least once with a diagnosis of CD	Nonmatching national registration numbers, other data irregularities. Authors excluded cancers occurring during the first year after entry into the cohort	diabetes mellitus, gastroenteritis, unspecified abdominal pain, anemia, ulcerative colitis, asthma, vertigo, constipation, pneumonia, congestive heart failure, failure to thrive, Down’s syndrome, rheumatoid arthritis, atrial fibrillation, angina pectoris	An elevated risk of PC (SIR, 1.9; 95% CI, 0.9–3.6) was observed. One of the PC patients had diabetes.
Card et al. 2004 [33] England	A population-based prospective cohort study	865/2	All age categories (0–60+) 444 F 193 M	Patients with the diagnosis of CD based on small bowel biopsy (severe or total VA as a result). The diagnosis date of patients diagnosed clinically in childhood but who did not have an intestinal biopsy until adult life was considered as the date of the intestinal biopsy.	Patients referred from other secondary care centers.	not reported	No overall increase in the rate of incident malignancy in patients with CD compared with the general population. No increase in the risk of GI carcinomas in general.
Goldacre et al. 2008 [34] England	A hospital-based retrospective cohort study	1997/2	All age categories (0–75+) Gender not Reported	Records of all hospital admissions with ICD codes relevant to CD	In the main findings, the data are shown excluding cancers in the first year after admission for CD	not reported	The overall risk of cancer was increased when the first-year cases were included and the increased risk was reduced when first year cases were excluded
Landgren et al. 2011 [35] USA	A hospital-based retrospective cohort study	63/13	18–100 Only M	Discharge diagnoses as defined by the 8th and 9th revisions of the International Classification of Diseases (ICDA, ICD9-CM)	No prior malignancy	Smoking-related diagnoses: emphysema, bronchitis, COPD excluding asthma, COPD including asthma hypertension, alcoholism, diabetes mellitus, obesity, HIV, hepatitis viral, hepatitis (acute, chronic), GERD infectious mononucleosis	The risk of PC was significantly increased (RR = 2.27 (95% CI, 1.22–4.23)
Elfstrom et al. 2012 [31] Sweden	Population-based cohort study	28,882/64	All age categories (0–60+) 17,893 F 10,989 M	Patients with the diagnosis of CD based on small bowel biopsy: 29,148 patients with CD diagnosis based on a small intestinal biopsy with VA, (equivalent to Marsh 3) and 13,446 individuals with inflammation but without VA (Marsh 1–2).	Individuals with the biopsy originated from the ileum, individuals without matched reference individuals, Diagnosis of GI cancer before biopsy and study entry.	not reported	Only in the first year of follow-up patients with CD (HR, 1.50; 95% CI, 1.33–1.68), inflammation (HR, 2.04; 95% CI, 1.80–2.32) and latent CD (HR, 2.06; 95% CI, 1.44–2.96) were at increased risk of GI cancer. The highest relative risks for GI cancer in patients with CD, inflammation and latent CD were seen for small intestinal cancer and PC (HR, 10.7; 95% CI, 5.77–19.7).
Ilus et al. 2014 [36] Finland	A population-based prospective cohort study	11,991/45	15–60+ 21,158 F 11,281 M	Duodenal biopsy showing typical VA with crypt hyperplasia	Cancers that occurred before entry to the registerwere excluded	DH	The SIR for malignancies was increased after 5 years from the diagnosis of CD. The SIRs for PC were decreased (0.73; 0.53–0.97). The risk was decreased especially in females (SIR = 0.59, 95% CI 0.36–0.89).
Koskinen et al. 2020 [37] Finland	Population-based cohort study	12,803/26	All age categories (0–60+) 7943 F 4860 M	Patients with the diagnosis of CD based on biopsy.	Inadequate baseline data	not reported	Mortality from all malignancies (HR 1.11, 95% CI 0.96–1.27) and GI malignancies (HR 1.21, 95% CI 0.56–1.71) was not increased among patients with CD.
Lebwohl et al. 2022 [30] Sweden	Population-based cohort study	47,241/152	0.0–95.4 29,381 F 17,860 M	Diagnosis of CD was defined as having relevant SnoMed codes corresponding to VA in the small intestine (other than the ileum) through 31 December 2016.	CD patients and controls with a record of cancer preceding the date of CD diagnosis (defined as the time of the first small intestinal biopsy showing VA) or the corresponding date for controls	type 1 diabetes, autoimmune thyroid disease, rheumatoid arthritis, and inflammatory bowel disease were more common in patients with CD than in controls	The overall risk of cancer was increased and was significantly elevated in the first year after CD diagnosis. The association between CD and cancer was noted among the others in PC (HR 2.30; 95% CI, 1.87–2.82). The elevated risk of PC persisted in long-term observation.

CD, celiac disease; PC, pancreatic cancer; NHL; non-Hodgkin lymphoma; GI, gastrointestinal; DH, dermatitis herpetiformis; GERD, gastroesophageal reflux disease; VA, villous atrophy; ICD, International Classification of Diseases; SnoMed, Systematized Nomenclature of Medicine; F, females; M, males; SIR, standardized incident ratio; HR, hazard ratio; RR, relative risk; CI, confidence interval.

**Table 3:** Analysis of the association between celiac disease and pancreatic cancer risk.

Author, Year	PC in CD *	CD *	PC in Patients without CD	Patients without CD	Total Number of Patients	%	RR (95% CI)	OR (95% CI)	*p* Value *
Askling et al., 2002 [32]	9	240	4	131	384	0.8	1.22 (0.38–3.89)	1.23 (0.37–4.06)	0.736
Goldacre et al., 2008 [34]	2	572	89	14,758	15,421	32.2	0.58 (0.14–2.35)	0.58 (0.14–2.36)	0.441
Landgren et al., 2011 [35]	13	50	535	7852	8450	17.6	3.23 (1.98–5.29)	3.82 (2.06–7.07)	0
Ilus et al., 2014 [36]	45	1581	62	1673	3361	7.0	0.775 (0.53–1.13)	0.77 (0.52–1.13)	0.218
Lebwohl et al., 2022 [30]	152	3735	302	16,136	20,325	42.4	2.17 (1.76–2.58)	2.17 (1.78–2.65)	0
Overall	221	6178	992	40,550	47,941	100.0	1.4 (1.25–1.67)	1.46 (1.26–1.7)	0

PC, pancreatic cancer; CD, celiac disease *;* RR relative risk; OR, odds ratio; CI, confidence interval; * Mantel-Haenszel test. *-patients with CD and malignancies.

## Data Availability

Not applicable.
